# Treatment of primary esophageal lymphomas: A review

**DOI:** 10.3389/fonc.2025.1475153

**Published:** 2025-02-06

**Authors:** Joe El Khoury, Remy Daou, Neal Kim, Josiane Bou Eid, Brandon Imber, Joachim Yahalom, Carla Hajj

**Affiliations:** ^1^ Department of Family Medicine, Saint Joseph University, Beirut, Lebanon; ^2^ Department of Medical Physics, Memorial Sloan Kettering Cancer Center, New York, NY, United States; ^3^ Department of Radiation Oncology, Memorial Sloan Kettering Cancer Center, New York, NY, United States; ^4^ Radiation Oncology, Oncology Institute, Cleveland Clinic Abu Dhabi, Abu Dhabi, United Arab Emirates

**Keywords:** esophageal lymphoma, DLBCL, follicular lymphoma, radiation treatment, MALT lymphoma

## Abstract

Primary esophageal lymphoma is a rare malignancy that is difficult to diagnose and treat. While there have been significant advances in understanding the pathogenesis, clinical features, and treatment options, there is a lack of consensus on the most effective treatment approach. This literature review provides a comprehensive overview of the use of available treatment options for primary esophageal lymphoma, including surgery, radiotherapy, and chemotherapy. The review also highlights the current knowledge gaps that need to be addressed through further research. While no single treatment modality has emerged as a clear front-runner, a combination of these treatments may be the most effective approach in managing primary esophageal lymphoma, tailored to the histological subtypes.

## 1 Introduction

Primary esophageal lymphoma is a rare malignancy that accounts for less than 1% of all esophageal neoplasms and only 0.3% of all primary non-Hodgkin’s lymphomas (1). This disease is challenging to diagnose and treat due to its nonspecific clinical presentation and variable histological features. As a result, the optimal management of primary esophageal lymphoma remains unclear, and there is no standard treatment approach. In recent years, there have been significant advances in the understanding of the pathogenesis, clinical features, and treatment options for primary esophageal lymphomas. However, the literature on this topic is limited, and there is a lack of consensus on the most effective treatment approach.

To date, various treatment modalities have been used, including surgery, radiotherapy, and chemotherapy. Each approach has its benefits and limitations, and the choice of treatment depends on the stage, histological type, and the patient’s overall health status. Given the rarity of this malignancy, there is a lack of large randomized controlled trials to establish the most effective treatment approach. As a result, the literature on the management of primary esophageal lymphoma consists primarily of case reports and small case series. In this literature review, we aimed to provide an overview of the use of available treatment options for primary esophageal lymphoma. We reviewed the existing evidence on the use of surgery, radiotherapy, chemotherapy, and immunotherapy and discuss their respective outcomes and limitations.

## 2 Search strategy

This literature review aimed to identify relevant studies related to primary esophageal lymphoma using the PubMed/Medline database. A comprehensive search was conducted using a combination of keywords and MeSH terms, including “primary esophageal lymphoma” OR “esophagus lymphoma” and various histology subtypes. No date or regional restrictions were applied, and all relevant analyses were included. The search yielded studies in several languages, and those in English, French, and Chinese were included in the analysis.

However, due to some studies being unavailable online, we only had limited access to their abstracts, resulting in some missing data. Despite this limitation, we included all available studies in our analysis to provide a comprehensive overview of the treatment options for primary esophageal lymphoma. The histological subtypes included in this study were MALT or mucosa-associated lymphoid tissue, DLBCL or diffuse large B-cell lymphoma, follicular lymphoma, Burkitt lymphoma, mantle cell lymphoma, T-cell lymphoma, ALCL or anaplastic large cell lymphoma, plasmablastic lymphoma, and Hodgkin lymphoma.

Overall, this review provides a comprehensive summary of the available literature on the management of primary esophageal lymphoma and highlights the current knowledge gaps that need to be addressed through further research.

## 3 Results

A total of 62 studies were included in this review. We found that most gastrointestinal tract lymphomas arise submucosally. They may be beyond the reach of endoscopic biopsy forceps, which can pose a problem for identifying histopathology and determining the best course of treatment (2). Reported reasons for choosing surgery, in indolent and less aggressive lymphomas like MALT lymphoma, included preliminary imaging, diagnosis, and a missing biopsy.

### 3.1 Mucosa-associated lymphoid tissue lymphoma

MALT lymphoma of the esophagus is a rare entity. In 2017, there were less than 20 reported cases of esophageal MALT lymphomas, most of which have been diagnosed in Japan (3). They are usually localized, but we found one case report of metastasis to the lungs and stomach (4) and one report of recurrence in the stomach and lung after initial radiotherapy (5). All treatment modalities have been used with generally good results, chemotherapy (CHOP-based) and resection being the most frequent (3, 4, 6–11). The main results of the available studies are summarized in **Table 1**.

**Table 1:** Treatment modalities and outcomes in primary esophageal MALT Lymphomas.

Initial treatment	Modality details or adjuvant treatment	Outcome	Follow-up range	2^nd^ line treatment	Number of patients	Reference
Surgery	Thoracoscopic-assisted resection with gastroesophageal anastomosis and jejunostomy	Complete remission	8 months	NA	1	(3)
	Left postero-lateral thoracotomy	Complete remission	1 year	NA	1	(43)
	+ Radiation (34Gy/17 Fractions) + eradication	Recurrence as metastasis after 21 months	21 months	CHOP	1	(5)
	Endoscopic mucosal resection	Complete remission	7-57 months	NA	4	(6, 44–46)
	Endoscopic mucosal resection + Radiation 30-36Gy	Complete remission	3-4 years	NA	2	(47, 48)
Chemotherapy	CVP	Complete remission	6 months	NA	1	(8)
	R-mini-CHOP	Partial remission	NA	NA	1	(9)
	Rituximab	Partial remission	1 year	NA	1	(10)
	R-CHOP + eradication	Complete remission, then sarcoidal granulomas	2 years	NA	1	(11)
Radiation therapy	36Gy/18 Fractions	Complete remission	9 months	NA	1	(49)
	36Gy 4 cycles + Rituximab	Complete remission	3 years	NA	1	(50)
Eradication therapy		Complete remission	3 years	NA	1	(51)

Despite the limited number of patients treated with radiotherapy in our review, extrapolation from other tumor sites would encourage the use of this modality, as MALT lymphomas are usually sensitive to radiation. In gastric lymphomas, radiotherapy can lead to 85-100% remission rates (4-5 years of disease-free survival), according to multiple small studies, especially in low-grade stages I and IIE (12–14). For this reason, since surgery—often indicated due to inconclusive biopsy results—and radiation therapy are effective treatment options with high remission rates, the safety profiles and tolerability of both modalities should be considered when treating patients with low-grade esophageal MALT lymphomas. Low doses of radiation therapy, compared to surgical resection, can be curative and associated with minimal side effects.

### 3.2 Follicular lymphoma

We found a single case of follicular lymphoma, described by Taal et al. in 1976. Based on the Working Formulation classification, it was diagnosed as a “small cleaved cell non-Hodgkin’s lymphoma” associated with extensive fistula formation, mainly to the left main bronchus. No manifestations of lymphoma were found outside of the esophagus. As dyspnea developed, the patient received a single dose of emergency chemotherapy but died three weeks later from sepsis (15).

### 3.3 Diffuse large B-cell lymphoma

DLBCL predominantly involves immunocompromised patients with HIV infection as a potential risk factor (16). All treatment modalities combined, outcomes were generally poor, and robust data is still lacking. While the results summarized in **Table 2** show a 50% complete response rate, in most case reports, follow-up duration was either lacking or was very short, thus masking early disease progression, which would have been attributed to treatment failure. However, combined modalities, including radiation therapy, seem to yield better results, as all 5 patients who received either chemotherapy plus radiation therapy or surgery followed by chemotherapy and radiation therapy had a complete response, with the reports covering a follow-up period of up to 3 years.

**Table 2:** Treatment modalities and outcomes in primary esophageal Diffuse Large B-Cell Lymphomas (DLBCL).

Initial treatment	Modality details or adjuvant treatment	Outcome	Follow-up range	2^nd^ line treatment	Number of patients	Reference
Radiation therapy	NA	NA	NA	NA	1	(52)
	(“large cell lymphoma”) 30Gy	Complete remission	1 month	NA	1	(52)
	NA, Palliative treatment	Death	1 month	NA	1	(20)
Chemotherapy	CHOP	Death during chemo	NA	NA	2	(53, 54)
	Nd-YAG laser, m-BACOD	Death from pneumonia	3 months		1	(20)
	R-CHOP (one patient stage 4)	Improvement	0-4 months	NA	3	(20, 55, 56)
	R-CHOP + Radiation	Complete remission	3 years	NA	2	(57, 58)
	Combination therapy	Complete remission	NA	NA	1	(59)
	R-CVP	Complete remission	NA	NA	1	(60)
	CEOP + radiation	Complete remission	3 years	NA	1	(61)
Surgery	Subtotal esophagectomy + R-CHOP + radiation	Complete remission	1 year	NA	1	(62)
No treatment	Patient refusal	Death, NA	6 weeks, NA	NA	2	(54, 62)

### 3.4 T-Cell lymphoma

Similarly to other types of esophageal lymphomas, T-cell lymphoma reports are scarce, and detailed treatment outcomes are missing in the literature. While two cases of primary NK/T-cell lymphoma were treated with chemotherapy only and achieved complete remission with a follow-up of up to 24 months (17, 18), a 40-year-old man in stage IE was also treated with CHOP but died due to complications (19).

Chemotherapy should be the mainstay of treatment, with a possible benefit when combined with radiation therapy. A review including Chinese case reports noted that systemic chemotherapy combined with concurrent radiotherapy might be an effective treatment for esophageal NK/T-cell lymphoma (17). Our literature search tends to validate this conclusion, as we noted a few cases of diffuse large T-cell Lymphomas treated with radiation therapy followed by either the HADAP regimen (patient survival of 14 months) or the CHOP regimen (2 patients, one with a survival of 7 months who died of sepsis despite evidence of early anti-tumor response, and one who survived ten months of follow-up) (20, 21). A fourth patient received two cycles of systemic chemotherapy with concurrent radiotherapy, but his general condition gradually worsened, and he passed away four months after admission (22).

Additionally, in 1999, 65 Gy irradiation was used to treat a primary esophageal T-cell lymphoma in clinical stage I(E)B with complete remission (23). The role of radiation therapy in T-cell lymphomas warrants further study, as radiotherapy has been considered of paramount importance for patients with clinically localized nasal disease, with approximately 70% of patients achieving complete remission after treatment (24).

### 3.5 Mantle cell lymphoma

Multifocal gut involvement has been described in mantle cell lymphoma (MCL) (25). However, esophageal involvement is uncommon. A retrospective study collecting data from 35 MCL patients with GI involvement found that the esophagus was affected in only 2 (5.7%) cases (26). All published cases were treated with chemotherapy, as lesions were usually seen in multiple locations in the GI tract. Of the few available cases, one died before receiving treatment (27); one was lost to follow-up, two had a good response to chemotherapy (28, 29), while one had no response to treatment (30). A “mantle cell lymphoma-like tumor” was treated with rituximab postoperatively and has been disease-free for more than 28 months after surgery (31).

### 3.6 Anaplastic large cell lymphoma

ALCL was first described in 1985 as a large-cell neoplasm with an expression of CD30 in all neoplastic cells (32). It is an uncommon T-cell lymphoma, defined as a separate entity since the 2008 WHO lymphoma classification (33). ALCL can be ALK+ or ALK-. Radiotherapy in esophageal ALCL has not yet been reported. Only one case report in 1992 mentioned a treatment plan including chemotherapy followed by radiotherapy, but the patient was lost to follow-up (34).

The few other published cases were treated with chemotherapy (usually CHOP), sometimes followed by autologous peripheral blood hematopoietic stem cell transplantation, with partial (2 cases) or complete (2 cases) remission (2, 35–37). Also, one patient was diagnosed post-mortem (38). Usually, ALK+ lymphoma is very sensitive to chemotherapy; thus, if the diagnosis is made without the need for thoracotomy and resection, chemoradiotherapy may be sufficient.

In other types and locations of ALCL, such as limited-stage systemic ALCL or breast implant-associated ALCL, radiation therapy was used in association with chemotherapy (39): for example, following doxorubicin-based chemotherapy and radiotherapy, in early-stage primary systemic anaplastic large-cell lymphoma, the 5-year overall survival (OS), progression-free survival (PFS), and local control rates for all patients were 84.4%, 63.6%, and 90.8%, respectively (40).

### 3.7 Burkitt

No case of adult esophageal Burkitt lymphoma was found in our database search. However, although it does not match our initial inclusion criteria, we found a case report of a 17-year-old patient in India with stage II intermediate risk, Group-B disease by Murphy staging system managed with LMB-96 protocol for intermediate-risk BL. She had a complete response and no recurrence after nine months (41).

### 3.8 Plasmablastic lymphoma

We found a single case of plasmablastic lymphoma treated in 2016 with resection followed by chemotherapy, without further details (42).

## 4 Discussion and conclusion

In conclusion, primary esophageal lymphoma, though rare, is a potentially life-threatening condition, requiring careful evaluation and management. The literature review explored various treatment options, including surgery, chemotherapy, and radiotherapy, with varying degrees of success. While no single treatment has emerged as a clear front-runner, a combination of those treatment modalities may be the most effective approach in managing primary esophageal lymphoma, and the choice should be tailored to the histological subtypes. Radiation therapy should be considered in patients with low-grade esophageal lymphomas as a definitive therapy, and as a consolidation therapy to chemotherapy in patients with high-grade esophageal lymphomas. Additional research is crucial to determine the most effective treatment strategies that could improve patient outcomes for this disease.
